# Effectiveness of a patient decision aid for women considering post-mastectomy breast reconstruction: randomized controlled trial

**DOI:** 10.1093/bjs/znaf151

**Published:** 2025-08-05

**Authors:** Britt A M Jansen, Isabelle J Henskens, Claudia A Bargon, Teun Teunis, Assa Braakenburg, Danny A Young-Afat, Helena M Verkooijen, Annemiek Doeksen

**Affiliations:** Department of Oncological Surgery, St. Antonius Hospital, Utrecht, The Netherlands; Department of Plastic, Reconstructive and Hand Surgery, St. Antonius Hospital, Utrecht, The Netherlands; Department of Oncological Surgery, St. Antonius Hospital, Utrecht, The Netherlands; Department of Plastic, Reconstructive and Hand Surgery, St. Antonius Hospital, Utrecht, The Netherlands; Department of Oncological Surgery, St. Antonius Hospital, Utrecht, The Netherlands; Department of Plastic, Reconstructive and Hand Surgery, St. Antonius Hospital, Utrecht, The Netherlands; Department of Plastic Surgery, University of Pittsburgh Medical Center, Pittsburgh, Pennsylvania, USA; Department of Plastic, Reconstructive and Hand Surgery, St. Antonius Hospital, Utrecht, The Netherlands; Department of Plastic, Reconstructive and Hand Surgery, Amsterdam University Medical Center, Amsterdam, The Netherlands; Division of Imaging and Oncology, University Medical Centre Utrecht, Cancer Centre, Utrecht, The Netherlands; Utrecht University, Utrecht, The Netherlands; Department of Oncological Surgery, St. Antonius Hospital, Utrecht, The Netherlands

## Abstract

**Background:**

Post-mastectomy breast reconstruction (PMBR) improves quality of life, yet decisions regarding type and timing are complex, requiring careful consideration of risks, benefits, and personal preferences. Shared decision-making enhances satisfaction with reconstructive decisions, but some women experience difficulty participating effectively. This study evaluated the effectiveness of a PMBR-specific patient decision aid (pDA) compared with high-quality standard care.

**Methods:**

In this single-centre RCT, women eligible for PMBR were assigned in a 1:1 ratio to either standard care alone (control) or standard care and a pDA (intervention). Questionnaires were completed at baseline, after reconstructive decision, and at 1-year post-surgery. The primary outcome was decisional conflict after the decision. Secondary outcomes included measures for the decision-making process, patient-reported, and surgical outcomes. Between-group differences were analysed using *t*-tests, Mann–Whitney U tests, and chi-square tests. Mean differences (MD) with 95% confidence intervals quantified outcomes, while effect sizes were determined by Cohen’s *d*.

**Results:**

A total of 134 patients were enrolled (66 intervention, 68 control). Both groups reported consistently low decisional conflict (17 and 20 respectively). No significant differences were found in the primary (MD −2.8, 95% c.i., −8.4 to 2.8, Cohen’s *d* −0.18) or secondary outcomes immediately after reconstructive decision or 1-year post-surgery.

**Conclusion:**

In a population with access to high-quality multidisciplinary care, an online pDA for PMBR did not provide additional benefits over standard care. Both approaches effectively supported reconstructive decision-making, resulting in consistently low levels of decisional conflict.

**Trial registration number:**

NL-OMON28003 (NTR), NL7939 (NTR-new)

## Teaser

This RCT evaluated whether an online patient decision aid provided additional benefits over high-quality standard care in supporting post-mastectomy breast reconstruction decisions. Among 120 women, both the pDA and standard care groups reported consistently low decisional conflict, with no significant differences in outcomes regarding the decision-making process, patient-reported outcomes, or surgical outcomes. This study suggests that in settings with high-quality multidisciplinary care, a pDA does not offer added benefits, as both strategies effectively support shared decision-making.

## Introduction

Women undergoing mastectomy increasingly opt for post-mastectomy breast reconstruction (PMBR) to improve quality of life (QoL), body image, and psychosocial well-being^1–4^. PMBR may use breast implants, autologous tissue, or a combination of both, and can be performed immediately or in a delayed surgical procedure^5^.

The decision-making process regarding type and timing of PMBR is complex, requiring careful consideration of surgical feasibility, logistical constraints, need and timing of (neo)adjuvant therapies, co-morbidities, body composition, and patient preferences^6,7^. Ideally, PMBR is a shared decision between the patient and multidisciplinary team, aligning oncological priorities with personal preferences to enhance long-term satisfaction with reconstructive outcomes^6–10^. Engaging in shared decision-making (SDM) can be challenging; women may experience difficulty understanding complex information and navigating choices, particularly following a recent breast cancer diagnosis, which can lead to decisional conflict and decisional regret^6,11^.

Patient decision aids (pDAs) support SDM by helping patients make informed choices that reflect their values, aiming to reduce decisional conflict while improving patient knowledge, satisfaction, and psychosocial well-being^11–14^. Decision-support tools specifically designed for PMBR however remain scarce, and it is unclear whether a PMBR-specific pDA offers benefits beyond high-quality standard care. In a setting of high-quality multidisciplinary care, an online PMBR-specific pDA tailored to the needs of Dutch women was developed and introduced one week prior to the patient’s initial consultation with a plastic surgeon.

An RCT was conducted to assess whether a pDA for PMBR reduces decisional conflict immediately after reconstructive decision compared to high-quality standard care. The secondary aim was to evaluate the impact of a pDA on the decision-making process, patient-reported outcomes (PROs), and surgical outcomes immediately after reconstructive decision and 1-year after surgery.

## Methods

### Design

A single-centre, two-arm, parallel group RCT was conducted at a large community teaching hospital in the Netherlands, where six plastic surgeons performed immediate and delayed PMBR. Patients were invited to participate between October 2019 and June 2023 by their oncological surgeon or nurse. After providing informed consent, participants were randomly assigned to a 1:1 ratio to the intervention or control group. Block randomization, with six patients per block, was used, with a computer-generated sequence concealed until assignment. The allocation sequence was independently generated and managed by an investigator (J.K.), who was not involved in patient enrolment, assignment, or data analysis. The control group received standard care alone and the intervention group received standard care and access to the pDA. Blinding of patients, plastic surgeons, and researchers after assignment was not performed due to practical constraints.

Questionnaires were administered to participants at T0—baseline, one week before their first consultation with the plastic surgeon, T1—immediately after reconstructive decision and T2—1-year post-surgery.

The trial was registered in the International Clinical Trial Registry Platform (ICTRP; NL-OMON28003). The study adhered to the Declaration of Helsinki (version 2013) and the Medical Research Involving Human Subjects Act, followed CONSORT guidelines, and was approved by the Medical Research Ethics Committee (MEC-U reg. no. W19.176)^15^.

### Participants

Women who were 18 years or older, required unilateral or bilateral mastectomy due to invasive or *in situ* breast cancer or due to prophylactic reasons, and were eligible for delayed or immediate PMBR. The patients had to have the ability to complete an online questionnaire and to comprehend the sixth-grade reading-level language used in the pDA and provide written informed consent. Women with prior breast reconstruction, those that already had discussed PMBR, had previously accessed a pDA for PMBR, or had metastatic disease were excluded. Following enrolment, some participants initially scheduled for mastectomy became eligible for and underwent breast-conserving surgery (BCS) after neoadjuvant systemic therapy. These participants were included in the analysis immediately after reconstructive decision (T1), reflecting real-world clinical decision-making, where treatment pathways may change after initial assessment and SDM. They were, however, excluded from the analysis at 1-year post-surgery (T2), as they did not undergo mastectomy or PMBR.

### Intervention

An online pDA for PMBR (https://www.keuzehulp.info/front-page/keuzehulpen/borstreconstructie) was developed using the Ottawa Decision Support Framework and International Patient Decision Aid Standards checklist^16^. Content was derived from local and international guidelines and co-created with decision aid experts, linguists, medical professionals, and patients. The Easy Reading Foundation (Stichting Makkelijk Lezen) ensured a sixth-grade reading level, optimizing accessibility for low health literacy^17^. The pDA was reviewed by members of the Dutch Breast Cancer Association (Borstkanker Vereniging Nederland)—patient representatives with lived experience of breast cancer and reconstruction. Their recommendations were incorporated into the pDA^18^. The pDA featured multiple modules providing a comprehensive overview as well as comparison of the different options for type and timing of PMBR, highlighting their benefits, disadvantages, associated risks, and expected outcomes. The pDA also addressed common misconceptions. Embedded close-ended questions checked real-time understanding of provided information. Several media types, including text, realistic photos, illustrations, and videos enhanced engagement. Value clarification exercises (visual-analogue scales, multiple choice and free-text questions) helped patients to align informed choices with personal values. A final summary consolidated personal preferences, expectations, and outstanding questions to facilitate SDM. The pDA takes approximately 15–20 min to complete and is updated every 2 years or whenever new guidelines are introduced to ensure integration of the best available evidence.

The intervention group was granted access to this online pDA one week before their first consultation with a plastic surgeon and patients were instructed to complete it beforehand. Completion status was monitored and reminders were issued to participants whose pDA remained incomplete prior to their initial appointment.

### Standard care

Both groups received high-quality multidisciplinary standard care. Prior to first consultation with the plastic surgeon, the oncological surgeon and/or nurse provided written information on the different types of PMBR, including pros, cons, potential complications as well as post-surgery guidelines and exercises through information leaflets. At the first consultation with the plastic surgeon, patients received a tailored verbal explanation, supplemented by photos and drawings of surgical PMBR options, and referral to a relevant website: BBewust (b-bewust.nl [in Dutch]) providing information and visual resources. When desired, additional consultations were scheduled with the plastic surgeon.

### Study measures

Following enrolment, participants completed an online questionnaire to collect baseline characteristics and responses to validated scales, including the Dutch General Self-Efficacy Scale (GSES), Hospital Anxiety and Depression Scale (HADS), and the preoperative BREAST-Q module. Clinical data regarding (neo-)adjuvant non-surgical treatments were retrieved from electronic medical patient records.

Outcomes domains included: (1) decision-making process, (2) PROs, and (3) surgical outcomes. The primary outcome was decisional conflict immediately after reconstructive decision (T1), assessed using the Decisional Conflict Scale (DCS)^19,20^. Decisional conflict was also assessed 1-year post-surgery (T2) as a secondary outcome. The decision-making process was further evaluated using, the SDM-Q-9 questionnaire to measure patients’ perceived involvement in SDM, the CARE questionnaire to assess patient-rated physician empathy, and the Decision Regret Scale (DRS) to assess decision regret^21–23^. Satisfaction with consultation(s) and information provided was measured using a study-specific questionnaire (0–10 scale). Duration of the first reconstructive consultation (in minutes) was measured by the researcher, and the number of consultations prior to reconstructive decision was documented. Satisfaction with plastic surgeon was measured using postoperative BREAST-Q and changes in the treating plastic surgeon were obtained through a study-specific questionnaire.

PROs included symptoms of anxiety and depression, measured with the HADS; satisfaction with breast(s), psychological well-being, sexual well-being, and physical well-being of the chest, evaluated using the postoperative BREAST-Q module; body image and breast symptoms, measured using the EORTC-QLQ-BR23 questionnaire; and overall satisfaction with treatment using a study-specific questionnaire (0–10 scale).

Surgical outcomes included the reconstructive choices (that is type and timing) and received breast surgeries, along with changes to surgical pathways (following initial reconstructive decisions). Data were collected through a study-specific questionnaire or obtained from electronic medical patient records when missing. A detailed overview of outcome measures and data collection is provided as Supplementary material (Appendix I, *Table S1*).

### Sample size

Manne *et al*. reported a mean decisional conflict score of 34 in patients considering PMBR^24^. An a priori sample size calculation determined that 57 patients per group would be needed to detect an eight-point difference in decisional conflict, assuming a decisional conflict score of 34 (s.d. 15) in the control group, with an alpha of 0.05 and power of 0.80. This corresponds to a medium effect size of 0.53. Allowing for a 5% loss to follow-up, a target sample size was set at 120 included participants (that is 60 participants per study arm).

### Statistical methods

Descriptive statistics were summarized as means with standard deviations for continuous variables and as counts with percentages for categorical variables. Between-group differences at each time point were evaluated using independent *t*-tests for normally distributed continuous variables, Mann–Whitney U tests for non-normally distributed continuous variables, and chi-square tests for categorical variables.

Mean differences with 95% confidence intervals quantified outcomes, whereas effect sizes were determined by Cohen’s *d* as follows: small (0.2), medium (0.5), and large (0.8). Clinically relevant differences were predefined as those with an effect size of 0.5 or greater^25^. All analyses adhered to the intention-to-treat principle and were performed using IBM SPSS Statistics Version 29 and R Version 4.4.0.

## Results

### Patients

**Fig. 1:**
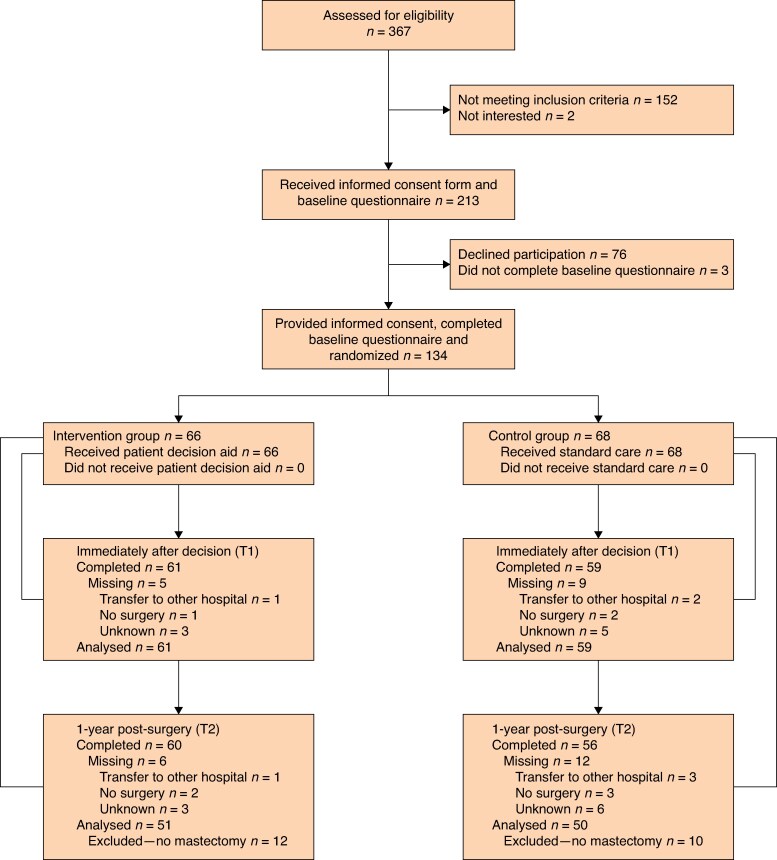
CONSORT flow diagram

Recruitment took place from August 2020 to May 2023, with follow-up completed in August 2024. Of 367 breast cancer patients assessed for eligibility, 213 were informed about the study. The main reasons for exclusion were choice of BCS, language barriers, and prior consultations with a plastic surgeon elsewhere. Informed consent was obtained from 134 participants, who completed the baseline questionnaire and were randomized to the intervention (*n* = 66) or control (*n* = 68) groups (*Fig. 1*). Reasons for declining participation included low interest in research, concurrent enrolment in other studies, or short notice to consider participation. All patients in the intervention group completed the pDA. Response rates were 90% immediately after reconstructive decision, with 120 patients eligible for primary outcome analysis, and 87% at 1-year post-surgery, without significant differences between groups (*Table S2*). Reasons for attrition included cancelled surgery (in palliative setting) or logistical transfers to other hospitals. All patients in the intervention group received and completed the pDA.

Baseline demographics and clinical characteristics were comparable between the two groups, indicating successful randomization (*Table 1*).

**Table 1:** Baseline characteristics of women eligible for post-mastectomy breast reconstruction randomized to a patient decision aid (pDA) or standard care

	pDA	Standard care
	(*n* = 66, 49%)	(*n* = 68, 51%)
*Patient characteristics*		
Age at enrolment (years), mean(s.d.)	50 (11.1)	50 (11.3)
BMI, median (i.q.r.)	24 (23, 29)	24 (22, 28)
**Co-morbidities per patient**		
0	54 (81.8)	46 (67.6)
1	12 (18.2)	19 (27.9)
2	0 (0.0)	2 (2.9)
3	0 (0.0)	1 (1.5)
**Preoperative cupsize**		
A	10 (15.2)	13 (19.1)
B	16 (24.2)	20 (29.4)
C	20 (30.3)	19 (27.9)
D	20 (30.3)	16 (23.5)
**Smoking status**		
Active smoker	7 (10.6)	2 (2.9)
Former smoker	19 (28.8)	31 (45.6)
Non-smoker	40 (60.6)	35 (51.5)
Education (years), median (i.q.r.)	17 (15, 19)	17 (15, 20)
**Marital status**		
Married	37 (56.1)	32 (47.1)
Living together, not married	17 (25.8)	18 (26.5)
Partner, not living together	2 (3.0)	3 (4.4)
No partner	7 (10.6)	13 (19.1)
Widow	3 (4.5)	1 (1.5)
Other	0 (0.0)	1 (1.5)
**Employment status**		
Employed	50 (76.9)	41 (60.3)
Unemployed	6 (9.2)	8 (11.8)
Retired	5 (7.7)	10 (14.7)
Other	4 (6.2)	9 (13.2)
*Baseline questionnaire scores*		
GSES, mean(s.d.)	33 (3.6)	33 (4.5)
**HADS, mean(s.d.)**		
Anxiety	6.6 (4.1)	7.0 (4.0)
Depression	3.8 (3.1)	3.9 (2.9)
**BREAST-Q, mean(s.d.)**		
Psychosocial well-being	66 (17)	69 (16)
Sexual well-being	62 (18)	58 (14)
Satisfaction with breasts	63 (18)	63 (19)
Physical well-being: chest	79 (19)	79 (18)
*Treatment characteristics*		
**Chemotherapy**		
Preoperative	23 (39.0)	20 (34.5)
Postoperative	14 (23.7)	11 (19.0)
Radiotherapy	24 (40.0)	19 (32.8)
**Other received non-surgical treatment**		
Endocrine and immunotherapy	5 (7.6)	6 (8.8)
Endocrine therapy	28 (42.4)	18 (26.5)
Immunotherapy	1 (1.5)	1 (1.5)
None	32 (48.5)	43 (63.2)

Values are presented as *n* (%) unless otherwise indicated. BREAST-Q = breast questionnaire; GSES = general self-efficacy scale; HADS = hospital anxiety and depression scale; pDA = patient decision aid.

### Primary outcome

Immediately after reconstructive decision, mean decisional conflict scores were 17 (s.d. 12) in the intervention group and 20 (s.d. 18) in the control group, without statically significant differences (mean difference (MD) = −2.8, 95% c.i., −8.4 to 2.8, *P* = 0.319) and a small effect size (Cohen’s *d* = −0.18; *Table 2*).

**Table 2:** Comparing decision-making and patient-reported outcomes after reconstructive decision and 1-year post-surgery in women eligible for post-mastectomy breast reconstruction randomized to a patient decision aid or standard care

	pDA		Standard care				
	*n*	Mean (s.d.)*	*n*	Mean (s.d.)*	MD (95% c.i.)	Cohen’s *d*	*P*
**Immediately after reconstructive decision**							
DCS	61	17 (12)	59	20 (18)	−2.8 (−8.4, 2.8)	−0.18	0.319
SDM-Q-9	60	83 (27)	59	83 (27)	−0.019 (−9.9, 9.8)	0.00	0.997
Patient-rated physician empathy (CARE)	61	38 (9.2)	59	37 (9.3)	1.1 (−2.3, 4.4)	0.12	0.527
Satisfaction with consultation(s)	61	9.6 (1.2)	59	9.5 (1.2)	0.15 (−0.3, 0.60)	0.13	0.487
Satisfaction with information	61	9.6 (1.1)	59	9.4 (1.2)	0.20 (−0.2, 0.60)	0.17	0.346
Duration of first consultation (minutes)	58	46 (15)	58	43 (15)	2.8 (−2.6, 8.2)	0.19	0.304
Number of consultations, *n* (%)	61		59				0.310
1		43 (70)		45 (76)			
2		17 (28)		11 (19)			
3		1 (2)		3 (5)			
Anxiety (HADS)	61	5.7 (3.7)	59	6.3 (3.9)	−0.60 (−2.0, 0.80)	−0.16	0.386
Depression (HADS)	61	3.9 (3.2)	59	3.8 (3.3)	0.071 (−1.1, 1.2)	0.020	0.905
**1-year post-surgery**							
Decisional conflict (DCS)	51	20 (22)	50	25 (22)	−4.9 (−14, 3.8)	−0.22	0.262
Decisional regret (DRS)	51	16 (16)	50	16 (21)	−0.51 (−7.9, 6.9)	−0.030	0.890
Satisfaction with information	49	9.3 (1.6)	49	9.0 (1.4)	0.35 (−0.27, 0.96)	0.23	0.266
Satisfaction with plastic surgeon (BREAST-Q)	50	93(12)	50	89 (15)	4.4 (−0.91, 9.8)	0.33	0.103
Change in treating plastic surgeon, *n* (%)†	44	0 (0)	39	1 (2) ‡			0.470
Anxiety (HADS)	50	5.1(3.9)	50	5.1(3.9)	0.06 (−1.5, 1.6)	0.02	0.938
Depression (HADS)	51	3 (3.6)	50	2.8 (3.2)	0.18 (−1.2, 1.5)	0.05	0.792
Satisfaction with breast(s) (BREAST-Q)	50	62 (14)	50	66 (20)	−3.8 (−11, 3.0)	−0.22	0.271
Psychosocial well-being (BREAST-Q)	50	68 (21)	50	69 (19)	−1.1 (−9.0, 6.7)	−0.06	0.778
Physical well-being: chest (BREAST-Q)	36	72 (22)	35	76 (17)	−3.6 (−13, 5.8)	−0.18	0.444
Sexual well-being (BREAST-Q)	49	55 (22)	48	53 (20)	2.0 (−6.5, 11)	0.10	0.635
Body image (EORTC-QLQ-BR23)	51	67 (24)	50	74 (26)	−6.7 (−16, 3.1)	−0.27	0.178
Breast symptoms (EORTC-QLQ-BR23)	51	70 (24)	50	78 (20)	−7.9 (−17, 0.82)	−0.36	0.075
Satisfaction with treatment	49	9.1 (1.9)	47	9.2 (1.1)	−0.13 (−0.76, 0.50)	−0.08	0.681

BREAST-Q = breast-questionnaire; CARE = consultation and relational empathy; DCS = decisional conflict scale; DRS = decisional regret scale; EORTC-QLQ-BR23 = European organization for research and treatment of cancer breast cancer-specific module; HADS = hospital anxiety and depression scale; pDA = patient decision aid; SDM-Q-9 = shared decision-making questionnaire. *Values are presented as mean(s.d.) unless otherwise indicated. †In patients who opted for reconstruction. ‡One patient wanted to consult a female plastic surgeon.

### Secondary outcomes

#### Decision-making process

Immediately after reconstructive decision, no significant differences were observed between groups in patients’ perceived involvement in SDM, patient-rated physician empathy or satisfaction with consultation and information, duration of the first consultation, or the number of consultations (*Table 2*).

At 1-year post-surgery, no significant differences were found in decisional conflict, decision regret, satisfaction with information, satisfaction with treatment, satisfaction with surgeon, or change in treating plastic surgeon (*Table 2*).

#### Patient-reported outcomes

Immediately after reconstructive decision, no significant differences were found between groups regarding symptoms of anxiety and depression.

At 1-year post-surgery, no significant differences were observed between intervention and control groups in symptoms of anxiety and depression, satisfaction with breast(s), psychological well-being, physical well-being of the chest, sexual well-being, body image, breast-related symptoms, or satisfaction with treatment (*Table 2*).

#### Surgical outcomes

There were no significant differences in actual reconstructive choices, including the decision to undergo reconstruction, as well as the type and timing of the procedure (*Table 3*). The majority of the participants underwent immediate PMBR, most commonly with autologous techniques. At 1-year post-surgery, no significant differences were observed in changes to surgical pathways between groups (*Table 3*).

**Table 3:** Comparing reconstructive choice, received breast surgery and changes in surgical pathways in women eligible for post-mastectomy breast reconstruction randomized to a patient decision aid or standard care

	pDA	Standard care	*P*
**Reconstructive choice**	*n* = 61	*n* = 59	0.483
*Immediate breast reconstruction*	50 (82)	42 (71)	
Autologous	36 (59)	33 (56)	
Implant-based	12 (20)	9 (15)	
Combined	2 (3)	0 (0)	
*Delayed breast reconstruction*	2 (3)	2 (3)	
Autologous	1 (2)	0 (0)	
Implant-based	1 (2)	2 (3)	
*No reconstruction*	9 (15)	15 (25)	
**Received breast surgery**	*n* = 51	*n* = 50	0.542
*Immediate breast reconstruction*	43 (84)	37 (74)	
Autologous	31 (61)	29 (58)	
Implant-based	10 (20)	8 (16)	
Combined technique	2 (4)	0 (0)	
*Delayed breast reconstruction*	1 (2)	3 (6)	
Autologous	0 (0)	0 (0)	
Implant-based	1 (2)	3 (6)	
*No reconstruction*	7 (14)	10 (20)	
**Change to surgical pathway**	*n* = 50	*n* = 50	0.240
Yes	2 (4)	1 (2)	
No	48 (96)	49 (98)	
Reason for change	*n* = 2	*n = 1*	
Patient's preference changed after peer support	1 (50)	0 (0)	
Preferred reconstruction not an option this patient	1 (50)	1 (100)	

Values are presented as *n* (%). pDA = patient decision aid.

## Discussion

In this RCT, the online pDA designed for women considering PMBR did not reduce decisional conflict. Both groups received high-quality standard care, resulting in consistently low levels of decisional conflict. No significant differences were observed immediately after reconstructive decision or 1-year post-surgery in any measure related to the decision-making process, PROs, or surgical outcomes.

Results diverged from the broader evidence supporting the effectiveness of pDAs in facilitating decision-making. A meta-analysis of 209 studies across various clinical contexts found that pDAs increased patient participation in decision-making^26^. pDA’s for oncological treatment of breast cancer, such as choices between BCS and mastectomy and/or decisions about systemic therapy—have been demonstrated to reduce decisional conflict while improving patient knowledge^11,27,28^. Patients considering PMBR however tend to be younger, more digitally literate, and are considering expected aesthetic results in addition to oncological outcomes, which may influence their information-seeking behaviour.

Specifically for PMBR, a recent network meta-analysis of 14 RCTs showed that pDA users experienced significantly less decisional conflict and regret, alongside improved knowledge and satisfaction^29^. The reported effect sizes were, however, small. Heterogeneity of the included studies, such as variations in pDA quality, timing of introduction (relative to consultations), delivery methods (for example web-based *versus* paper-based), outcomes measures, and type of PMBR performed (for example primarily delayed *versus* immediate), complicated direct comparisons with the present study.

In a study by Sherman *et al*., pDA users reported significantly less decisional conflict one month after randomization (MD = −8.3), but their study lacked standardization of information provided to control groups across different study sites and had low participant numbers per site (<30) resulting in practice variation^30^. Mean DCS scores in their control group at 1- and 6-month follow-up (that is 36 and 31 respectively) were considerably higher than the DCS scores observed in the present control group immediately after reconstructive decision and at 1 year after surgery (that is 20 and 25 respectively). Although timelines do not align, substantial differences in decisional conflict highlight the extent to which the control group in the present study was well-informed and supported. Their cohort primarily focused on delayed PMBR (54%), limiting the applicability of their finding to the present setting, in which immediate PMBR was more performed. Additionally, their study was prone to self-selection bias due to a high dropout rate (27%) of participants having greater social support. In contrast, the current trial experienced only 13% attrition at one-year follow-up. Known reasons for attrition and declining participations were likely unrelated to key concepts such as decision-making confidence, self-efficacy, social support, anxiety or information-seeking behaviour, thereby mitigating the risk of selection bias in the current trial.

In this study, decisional conflict was low in both groups (that is ≤25), indicating that women were generally ready to implement a decision^19^. The low decisional conflict in the control group suggests that the standard care provided at the study institution effectively met patient decision-making needs. Women in both groups reported high satisfaction with the information provided and felt well-supported in their decision-making, likely diminishing the potential effect of the pDA. This comprehensive care left minimal room for further improvement and likely diminished the potential effect of the pDA. Key components of the high-level decisional support include: close collaboration among the multidisciplinary team (that is plastic surgeons, oncologic surgeons, radiotherapists, oncologists, and physician assistants), oncological surgeons and physician assistants dedicating time prior to the consultation with plastic surgeon to inform patients regarding reconstructive options, structured information leaflets with links to websites and visual resources to ensure patients have access to accurate resources in multiple formats, and individualized decisional support during consultations. Yet, study enrolment might have heightened awareness for involvement in shared-decision making and encouraged greater information-seeking behaviour in the control group, also known as the Hawthorne effect^31^.

The findings of this trial align with those of Ter Stege *et al*. and Politi *et al*., both of which did not observe significant differences in decision conflict, SDM, or QoL between web-based pDA users and those receiving enhanced standard care^32,33^. Ter Stege *et al*. conducted an RCT in women considering immediate PMBR comparing an online pDA with enhanced standard care, which included actively providing a typical Dutch information leaflet^32^. Similarly, Politi *et al*. evaluated a web-based pDA compared to enhanced usual care, where participants received the American Society of Plastic Surgeons pamphlet about PMBR^33^. Although improvements were reported in patient knowledge and preparedness, this did not translate into reduced decisional conflict. These findings suggests that although pDAs may enhance patients’ knowledge, they might not outperform standard care in reducing decisional conflict in already well-supported settings. Although objective knowledge was not assessed as a separate outcome measure in the present study, the pDA included close-ended questions to confirm real-time understanding of information, and perceived comprehension was measured by the DCS ‘informed’ subscale as part of the primary outcome assessment. Both groups scored similarly low on this subscale (mean 18 in the pDA group *versus* 19 in the control group), with a lower score depicting stronger perceived understanding, indicating that both groups felt equally well informed (data not shown). Another explanation for the findings of the current study is that the pDA may not have been optimally designed. If the pDA did not deliver information in a sufficiently engaging or accessible format, patients may not have used it in a way that meaningfully impacted their decision-making. Wilkens *et al*. however reported a reduction in decisional conflict when using a comparable pDA that followed the same guidelines for development for decisions regarding trapeziometacarpal arthritis^34^. Similarly, Mardinger *et al*. compared two pDAs with varying levels of interactivity and found that both pDAs equally reduced decisional conflict over time^35^. Their study was, however, conducted in a low-volume centre, where the pDA was provided between two consultations with the same plastic surgeon. In contrast, the current trial was conducted in a high-volume, multisurgeon environment, with the pDA provided before the first consultation. Additionally, their study lacked a control group, so the observed trend in decisional conflict may reflect the natural course of decisional conflict over time.

Apart from patients, healthcare professionals may also benefit from offering pDAs. In a qualitative study, Sherman *et al*. found that, from a health professionals’ perspective, pDAs could help patients prepare for medical appointments, thereby making consultations more efficient^36^. Despite this, the current study found no difference in consultation length with the use of the pDA, consistent with findings from previous research^26^. Nonetheless, pDAs could still reduce unwanted practice variation in the SDM process among healthcare professionals by standardizing patient information^37^.

A key strength of this study is its randomized controlled design and high response rates. However, a limitation is that one of the plastic surgeons was involved in creating and updating information for the pDA. Although the pDA was approved by all participating plastic and oncologic surgeons, this involvement may have influenced them to prime their standard of care regarding decisional support. Practical constraints prevented blinding of plastic surgeons to treatment allocation. No plastic surgeon-dependent differences were however found in exploratory analysis of the primary outcome regardless of treatment allocation (data not shown).

Shared decision-making is indispensable to PMBR given the inherently complex and profoundly personal nature of choices involved. This RCT found that, within a setting of high-quality standard care, the addition of an online patient decision aid did not further reduce decisional conflict. Both approaches consistently achieved low levels of decisional conflict, suggesting they were equally effective in supporting informed decision-making. In an era focused on the continuous enhancement of patient engagement, these findings highlight that context matters: in well-resourced multidisciplinary settings, standard care alone can effectively provide decisional support regarding PMBR.

## Supplementary Material

znaf151_Supplementary_Data

## Data Availability

Data are available on request from the corresponding author.
