# Supplementary material for: Effectiveness of a patient decision aid for women considering post-mastectomy breast reconstruction: randomized controlled trial
Source: Br J Surg. 2025 Aug 5;112(8):znaf151. doi: 10.1093/bjs/znaf151 (PMC12485225; doi:10.1093/bjs/znaf151)
Supplement: znaf151_Supplementary_Data [file znaf151_supplementary_data.docx]

**Title: Effectiveness of a patient decision aid for women considering post-mastectomy breast reconstruction: A randomized controlled trial**

**Authors**
Britt A.M. **Jansen**^1,2^, Isabelle J. **Henskens**^1,2^, Claudia A. **Bargon**^1,2^, Teun **Teunis** T^3^, Assa **Braakenburg**^2^, Danny A. **Young-Afat**^4^, Helena M. **Verkooijen**^5, 6^, Annemiek **Doeksen**^1^.

**Affiliations**

1. Department of Oncological Surgery, St. Antonius Hospital, Utrecht, The Netherlands

2. Department of Plastic, Reconstructive and Hand Surgery, St. Antonius Hospital, Utrecht, The Netherlands.

3. Department of Plastic Surgery, University of Pittsburgh Medical Center, Pittsburgh, Pennsylvania, United States of America.

4. Department of Plastic, Reconstructive and Hand Surgery, Amsterdam University Medical Center, Amsterdam, The Netherlands.

5. Division of Imaging and Oncology, University Medical Centre Utrecht, Cancer Centre, Utrecht, The Netherlands.

6. Utrecht University, Utrecht, The Netherlands.

**Corresponding Author**

B.A.M. Jansen, MD

Department of Oncological Surgery, St. Antonius Hospital

Department of Plastic, Reconstructive and Hand Surgery, St. Antonius Hospital

Soestwetering 1, 3543 AZ, Utrecht, The Netherlands

E-mail: [br.jansen@antoniusziekenhuis.nl](mailto:br.jansen@antoniusziekenhuis.nl)

ORCID ID: 0000-0002-7533-4932

**Supplementary Materials - Index**

| **Supplementary Appendixes** |  |
| --- | --- |
| **Appendix I.** Study measures | *page 3* |
| **Supplementary Figures and Tables** |  |
| **Table S1**. Outcomes and data collection immediately after reconstructive decision (T1) and 1-year post-surgery (T2) | *page 5* |
| **Table S2**. Response rates | *page 5* |
| **References** | *page 6* |
|  |  |

**Supplementary Appendixes**

**Appendix I:** Study measures

The DCS is a validated 16-item questionnaire that evaluates patients’ uncertainty during medical decision-making. Responses are scored on a 5-point Likert scale and total scores range from 0 to 100, with lower scores indicating less decisional conflict. Following the user manual, scores below 25 were associated with decision implementation, whereas scores above 37.5 were associated with decision delay and uncertainty.^1^

The General Self-Efficacy Scale (GSES) measures the ability to cope with a difficult situation.^2^ The GSES consists of 10 items, rated on a 4-point Likert scale (from 1 “not at all true” to 4 “exactly true”), and includes statements such as: “I can usually handle whatever comes my way”. The final score sums all the items and ranges from 10 to 40, with higher scores reflecting greater levels of self-efficacy.

The Hospital Anxiety and Depression Scale (HADS) measures levels of anxiety and depression in individuals with physical health conditions.^3^ It consists of 14 items divided into two subscales, each rated on a 4-point Likert scale (from 0 “Not at all” to 3 “Most of the time”), with options reflecting symptom frequency or intensity. Total score is the sum of scores and ranges from 0 to 21, with higher scores indicating greater severity of symptoms.

The pre- and post-operative BREAST-Q Module was employed, as it has become the gold standard for assessing PROs in breast surgery.^4^ The BREAST-Q assesses two overarching domains: patient satisfaction and health-related quality of life (HR-QoL). The subscales evaluated included psychological well-being, physical wellbeing of chest, sexual wellbeing and satisfaction with breast(s) and plastic surgeon for mastectomy alone and post-mastectomy reconstruction. Items are scored on a 3-, 4-, or 5-point Likert scale with options reflecting agreement, frequency, and satisfaction. Raw subscale scores are converted to a score ranging from 0 to 100, with higher scores are indicating better outcomes.

The 9-item Shared Decision-Making Questionnaire (SDM-Q-9) assesses the extent to which patients feel involved in the shared decision making (SDM) process.^5^ Each item addresses a specific step in SDM and is rated on a 6-point Likert scale, ranging from 0 (“completely disagree”) to 5 (“completely agree”). The sum scores are linearly transformed into a percentage of the maximum score, where 0 represents no patient involvement and 100 represents maximum involvement in SDM.

The Consultation And Relational Empathy (CARE) questionnaire is designed to capture patient-rated physician empathy across 10 items.^6^ These items assess how well physicians understand and respond to patients’ concerns and fears on a 5-point Likert scale from 1 (“poor”) to 5 (“excellent”). The final score sums items and ranges from 10 to 50, with higher scores reflecting a greater perceived level of physician empathy.

The European Organization for Research and Treatment of Cancer Breast Cancer-specific Module (EORTC-QLQ-BR23) includes subscales for body image and breast symptoms.^7^ The items are rated on a 4-point Likert scale ranging from 1 (“not at all”) to 4 (“very much”). A final score for each subscale is calculated on a scale from 0 to 100. For body image higher scores indicate better outcomes, while for breast symptoms lower scores reflect better outcomes.

The Decision Regret Scale (DRS) is a 5-item tool to measure regret after a healthcare decision. Items are rated on a 5-point Likert scale ranging from 1 (“strongly agree”) to 5 (“strongly disagree”). Scores are summed and converted to a scale ranging from 0 to 100, with 0 indicating no regret and 100 indicating high regret.^8^

Satisfaction with the visit, information, and overall treatment was assessed using three questions rated on a 0–10 scale, where 0 represented the worst and 10 the best possible outcome. Participants were asked: “How would you rate your overall satisfaction with today’s visit for your breast reconstruction?”, “How would you rate your overall satisfaction with the information provided today for your breast reconstruction?”, and “How would you rate your satisfaction with the treatment for your breast reconstruction?”

**Supplementary Figures and Tables**

| **Table S1.** Data collection of outcomes immediately after reconstructive decision (T1) and 1-year post-surgery (T2). | | |
| --- | --- | --- |
| **Outcomes (instrument)** | **T1** | **T2** |
| **Decision-making process** | | |
| Decisional conflict (DCS) | X | X |
| Shared decision making (SDM-Q-9) | X |  |
| Patient-rated physician empathy (CARE) | X |  |
| Satisfaction with consultation(s) (study-specific questionnaire) | X |  |
| Satisfaction with information (study-specific questionnaire) | X | X |
| Duration of first consultation (electronic patient file) | X |  |
| Number of consultations (study-specific questionnaire) | X |  |
| Decision regret (DRS) |  | X |
| Change in plastic surgeon (study-specific questionnaire) |  | X |
| Satisfaction with plastic surgeon (BREAST-Q) |  | X |
| **Patient-reported outcomes** | | |
| Anxiety and depression (HADS) | X | X |
| Satisfaction and QoL (post-operative BREAST-Q) |  | X |
| Body image and breast symptoms (EORTC QLQ-BR23) |  | X |
| Satisfaction with treatment (study-specific questionnaire) |  | X |
| **Surgical outcomes** | | |
| Reconstructive choice (study-specific questionnaire) | X |  |
| Received breast surgery (electronic patient file) |  | X |
| Change in surgical pathway (study-specific questionnaire) |  | X |

| **Table S2.** Response rates. |  |  |  |
| --- | --- | --- | --- |
|  | **Patient decision aid**  n (%) | **Standard care**  n (%) | p-value |
| Enrollment | 66/66 (100) | 68/68 (100) |  |
| Immediately after reconstructive decision | 61/66 (92) | 59/68 (87) | 0.40 |
| 1-year post-surgery | 60/66 (91) | 56/68 (82) | 0.21 |

**References**

1. O'Connor AM. User manual: Decisional Conflict Scale [document on the Internet]. Ottawa Hospital Research Institute. <https://decisionaid.ohri.ca/docs/develop/User_Manuals/UM_Decisional_Conflict.pdf>. Published 1993. Accessed2024.

2. Schwarzer R, Jerusalem M, Juczyński Z. The general self-efficacy scale (GSE). *Anxiety, Stress, and Coping.* 2009;12:329-345.

3. Spinhoven P, Ormel J, Sloekers P, Kempen G, Speckens AE, van Hemert AM. A validation study of the Hospital Anxiety and Depression Scale (HADS) in different groups of Dutch subjects. *Psychological medicine.* 1997;27(2):363-370. doi:<https://dx.doi.org/10.1017/S0033291796004382>.

4. Pusic AL, Klassen AF, Scott AM, Klok JA, Cordeiro PG, Cano SJ. Development of a new patient-reported outcome measure for breast surgery: the BREAST-Q. *Plastic and reconstructive surgery.* 2009;124(2):345-353. doi:<https://dx.doi.org/10.1097/PRS.0b013e3181aee807>.

5. Kriston L, Scholl I, Hölzel L, Simon D, Loh A, Härter M. The 9-item Shared Decision Making Questionnaire (SDM-Q-9). Development and psychometric properties in a primary care sample. *Patient education and counseling.* 2010;80(1):94-99. doi:<https://dx.doi.org/10.1016/j.pec.2009.09.034>.

6. Mercer SW, Maxwell M, Heaney D, Watt GC. The consultation and relational empathy (CARE) measure: development and preliminary validation and reliability of an empathy-based consultation process measure. *Family practice.* 2004;21(6):699-705. doi:<https://dx.doi.org/10.1093/fampra/cmh621>.

7. Aaronson NK, Ahmedzai S, Bergman B, et al. The European Organization for Research and Treatment of Cancer QLQ-C30: a quality-of-life instrument for use in international clinical trials in oncology. *JNCI: Journal of the National Cancer Institute.* 1993;85(5):365-376. doi:<https://dx.doi.org/10.1093/jnci/85.5.365>.

8. Brehaut JC, O'Connor AM, Wood TJ, et al. Validation of a decision regret scale. *Medical decision making.* 2003;23(4):281-292. doi:<https://dx.doi.org/10.1177/0272989X03256005>.
